# Relationship Satisfaction Among Autistic Populations: How Partner Neurotype Influences Relationship Satisfaction Factors for Autistic Adults

**DOI:** 10.1089/aut.2024.0124

**Published:** 2025-01-15

**Authors:** Jessica Khaw, Ty Vernon

**Affiliations:** University of California, Santa Barbara, California, USA.

**Keywords:** autistic, autism and relationships, relationship satisfaction, romantic relationships, communication, neurodivergent relationships

## Abstract

**Background:**

Autistic individuals report high levels of interest in pursuing and maintaining romantic relationships but indicate fewer and less satisfying relationships when compared with non-autistic populations. In this mixed-methods study, we investigated the self-identified factors that contribute to successful romantic relationships for autistic adults with the additional goal of understanding if and how their partners’ neurotypes may influence relationship satisfaction.

**Methods:**

We compared levels of relationship satisfaction among 106 autistic individuals by categorizing each participant into one of three groups based on the specific neurotype of their partner: (1) autistic/autistic (A/A) dyads; (2) autistic/neurotypical (A/NT) dyads; and (3) autistic/non-autistic but neurodivergent (A/ND) dyads. All participants completed a series of online surveys to gather information about their autistic traits, overall relationship satisfaction, reciprocal communication and support, and perceived impact of autism on relationships.

**Results:**

Across the three dyads, participants reported similar levels of relationship satisfaction, and we found no statistically significant differences when comparing the mean satisfaction of each sample group. Participants indicated a wide variety of positive and negative relational aspects, with a few themes changing in frequency based on partner neurotype. Participants with autistic partners tended to focus on an inherent and deep sense of understanding when discussing the positive aspects of their relationships, while those with non-autistic partners were more likely to discuss the presence of mutual support and accommodation. In addition to some differences in reported themes, participants tended to report that their autistic traits positively impacted satisfaction in A/A dyads, while those in A/NT and A/ND dyads tended to report that their autistic traits posed challenges in their relationships.

**Conclusions:**

The results of this study indicate that the neurotype of an autistic individual’s partner may influence some of the reported positive and challenging aspects of their relationships. However, survey results indicate that autistic populations generally find comparable levels of satisfaction in romantic relationships irrespective of neurotype, which suggests that while partner neurotype may shape the enabling and barring factors for relationship success, it does not directly affect the overall health of a relationship. Finally, how an autistic individual perceives the impact of autism on their relationship may be affected by the neurotype of their partner.

**Community Brief:**

**Why is this an important issue?**

Many autistic folks seek out and thrive in romantic relationships, but studies report that autistic people experience lower relationship satisfaction than non-autistic people. Despite this, the autism research field has made little progress toward understanding (a) why this is the case and (b) how we can better support autistic individuals who are navigating romantic relationships. To best understand what autistic people seek in relationships, we created a space where autistic participants could share their experiences without comparison with those of non-autistic people.

**What was the purpose of this study?**

We contributed to the current body of literature by learning more about what autistic people seek in relationships. We also wanted to see what they may struggle with and if these factors are influenced by whether their partner is neurotypical (referring to someone whose brain works in a way that is socially “typical”), neurodivergent (broadly referring to someone whose brain works differently from what is considered “typical”), and/or autistic. In doing this, we hope to help develop more effective ways of supporting autistic individuals who seek romantic connections.

**What did the researchers do?**

The lead researcher, J.K., developed the project based on her experiences navigating romantic relationships as an autistic person. She recruited 106 autistic participants to complete a series of surveys regarding relationship satisfaction. Then, participants completed free-response questions about the most positive and negative parts of those relationships. She asked how they felt autism affected their relationships and gathered these components to identify what increased and decreased relationship satisfaction.

**What were the results of the study?**

We found that autistic people are similarly satisfied in their relationships regardless of whether their partners are autistic or not. Despite this, participants consistently identified different positive and challenging factors within their relationships that tended to align with the neurotype of their partners. We found an interesting link between how autistic people view positive and negative factors in successful romantic relationships and their partners’ neurotypes.

**What do these findings add to what was already known?**

We added to a broad body of knowledge about relationship satisfaction by including a greater, more detailed consideration of autistic adults. Instead of comparing survey responses between autistic and non-autistic participants, we compared how autistic people viewed relationship satisfaction based on if their partners were autistic, non-autistic but still neurodivergent, or neurotypical.

**What are potential weaknesses in the study?**

Potential limitations include the following: (1) we recruited a low number of male-identified participants, (2) we did not collect co-occurring diagnoses and conditions from the autistic participants, and (3) we did not verify partner neurotype.

**How will these findings help autistic adults now or in the future?**

Through this project, we were able to highlight positive communication and interpersonal skills among autistic adults. We hope that the results of this study will help autistic people and their partners find some peace and community by reading about others with similar relationship experiences. We also hope to help them further understand what factors might serve as potential strengths and weaknesses in their relationships.

## Background

Existing literature focuses on how autism creates enablers and barriers to successful romantic relationships by comparing the experiences and perspectives of autistic and non-autistic individuals^1^ without considering the role of the partner’s neurotype. Previous investigations indicate that autistic individuals may find greater satisfaction with autistic partners,^2^ but these did not distinguish between participants with neurotypical and non-autistic neurodivergent (i.e., attention-deficit/hyperactivity disorder [ADHD], obsessive-compulsive disorder, borderline personality disorder) partners. Because relationships are bidirectional and transactional, it is crucial to examine how the neurotype of one’s partner might influence their perceived relationship satisfaction. In addition, each neurotype brings a unique set of spoken and unspoken preferences, behavioral tendencies, and communication styles, which must be explored to clarify how they align or diverge with partner expectations.

Current research suggests that common characteristics associated with autism can lead to challenges when pursuing and maintaining romantic relationships.^1^ The defining vulnerabilities in social communication and social–emotional reciprocity among autistic individuals have created the misconception that these individuals are less inclined to pursue romantic relationships than their non-autistic counterparts. This misconception has since been disproven,^2,3^ as autistic individuals demonstrate comparable levels of interest in romantic relationships as their non-autistic peers even if they are less likely to be in a relationship.^3,4^

There are many possible explanations for the lower likelihood of romantic engagement. Autistic individuals tend to have less regular social contact with peers, thus reducing opportunities to be around others and identify potential romantic partners. Autistic adults often report feeling socially isolated, with more than half consistently indicating an absence of close social relationships.^5–8^ They may also lack experience with or education in appropriate ways to initiate social contact due to challenges with social access.^8,9^ In addition, subtle or indirect methods (e.g., flirting) used and expected by neurotypical populations do not align with the autistic tendency to be straightforward with interests and intentions. Many autistic adults find it difficult to identify and reciprocate common flirting techniques, as they will often over-assume romantic intent from others^10^ and struggle to engage in conventional verbal and nonverbal communication strategies that indicate romantic interest.^11^ Mental health may also contribute to these differences, as there is a strong link between good mental well-being and willingness to initiate romantic relationships.^12^ Autistic individuals are four times more likely to develop depressive disorders when compared with neurotypical populations,^13^ and it is estimated that 42% of autistic individuals will develop co-occurring anxiety disorders throughout their lives.^14^ These high co-occurrences can directly impact the active pursuit of a romantic connection.

Both verbal communication and nonverbal communication are strongly and positively correlated with relationship satisfaction and success.^15^ In turn, relationship length and quality both contribute to overall happiness, with some research going so far as to label involvement in romantic relationships as an essential factor for maintaining happiness across the lifespan.^16,17^ The discrepancy between romantic desire and actual involvement raises questions about how differences in interpersonal communication style may affect the success of the romantic pursuits of autistic people as they tend to report lower levels of relationship satisfaction overall when compared with non-autistic partners.^1,3,4^ Given that divergence from neurotypical social communication norms is required for an autism diagnosis, it is important to increase our understanding of what defines a successful or unsuccessful relationship to include the lived experience of those who may have an alternative approach to interpersonal communication.

We expanded upon existing dating research by examining factors that contribute to relationship satisfaction for autistic individuals who are romantically involved with autistic, non-autistic neurodivergent, and neurotypical partners. We compared results across all three participant groups to understand how relationship dynamics change for autistic populations based on the neurotype of their partners. We hoped to determine what facilitates and hinders relationship satisfaction and success for autistic adults to explore if the neurotype of an autistic individual’s partner affects the overall satisfaction of the relationship. We also aimed to understand whether partner neurotype influenced the perceived impact of autism characteristics on the relationship.

Although there is variability in preference for identity-first language (“autistic person”) and person-first language (“person with autism”), identity-first language is currently more widely encouraged by the autistic community.^18,19^ In this article, we use “autistic” when appropriate; other neurotypes, such as neurotypical, will be specified throughout. In addition, it is important to acknowledge that the purpose of this study is not to “correct” or “fix” potential differences in how autistic individuals choose to conduct their romantic relationships. Rather, our goal is to gain a better understanding of how they define successful relationships without defaulting to neurotypical relationships as the standard.

## Methods

### Ethics approval

This study was approved by UC Santa Barbara’s Human Subject Committee. All participants completed an informed consent process before completing any portion of this study.

### Participants

The sample we used consisted of 106 U.S. residents. We recruited participants through various social media outlets (i.e., Facebook, Instagram) and online forums (i.e., Reddit). Inclusionary criteria consisted of the following: (1) a professional diagnosis or self-diagnosis of autism; (2) current or previous romantic relationship experience; and (3) scoring higher than the minimum cutoff score (>59) on the Social Responsiveness Scale, Second Edition (SRS-2). We asked eligible participants to complete an online Qualtrics survey consisting of demographic, quantitative, and qualitative components.

We compared levels of relational satisfaction among autistic individuals categorized into groups based on the neurotype of their most recent romantic partner: (1) autistic/autistic (A/A) dyads (*n* = 32, 30.19%); (2) autistic/non-autistic but neurodivergent (A/ND) dyads (*n* = 46, 42.99%); and (3) autistic/neurotypical (A/NT) dyads (*n* = 28, 26.42%). The majority of the participants were in a relationship (*n* = 89, 83.96%), and a small portion were not in a relationship but had prior relationship experience (*n* = 17, 16.04%). Just over half of the participants were professionally diagnosed with autism (*n* = 60, 57.01%); the rest were self-diagnosed (*n* = 46, 42.99%). We included self-diagnosed autistic individuals in this investigation to maximize the number of eligible participants and account for the widespread lack of adult access to autism-specific evaluation services. We required all participants to have SRS-2 total scores in the autism range. Participant demographics are summarized in Table 1.

**Table 1.** Sociodemographic Characteristics of Participants

Dyads	A/A		A/ND		A/NT		Total sample	
	*n*	%	*n*	%	*n*	%	*n*	%
Total	32	30.19	46	42.99	28	26.42	106	100
Age								
18–23	7	21.88	10	21.74	6	21.43	23	21.70
24–29	11	34.38	14	30.43	7	25.00	32	30.19
30–34	6	18.75	10	21.74	3	10.71	19	17.92
35–39	3	9.38	6	13.04	6	21.43	15	14.15
40–44	1	3.13	4	8.70	4	14.29	9	8.49
45–49	1	3.13	1	2.17	1	3.57	3	2.83
50+	3	9.38	1	2.17	1	3.57	5	4.72
Gender								
Male	6	18.75	8	17.39	4	14.29	18	16.98
Female	13	40.63	15	32.61	15	53.57	43	40.57
Non-binary	12	37.50	18	39.13	8	28.57	38	35.85
Other	1	3.13	5	10.87	1	3.57	7	6.60
Sexual orientation								
Heterosexual	5	15.63	8	17.39	7	25.00	20	18.87
Gay/lesbian	5	18.75	9	19.57	2	7.14	17	16.04
Bisexual	12	37.50	18	39.13	13	46.43	43	40.57
Other	9	28.12	11	23.91	6	21.43	26	24.53
Relationship status								
Current	28	87.50	39	84.78	22	78.57	89	83.96
Past	4	12.50	7	15.22	6	21.43	17	16.04

A/A, autistic/autistic dyads; A/ND, autistic/non-autistic but neurodivergent dyads; A/NT, autistic/neurotypical dyads.

### Data collection

#### The Social Responsiveness Scale, Second Edition

To support the inclusion of self-diagnosed participants, we administered the SRS-2 to each participant. The SRS-2 is a 65-item questionnaire measuring autistic characteristics across five subscales: social motivation, social awareness, social cognition, social communication, and restricted interests and repetitive behaviors (RRBs).^20^ This diagnostic aide, while not intended to serve as a primary diagnostic tool, identifies and quantifies the degree of multiple social difficulties commonly associated with autism. The SRS-2 has high predictive validity and a sensitivity value of 0.92.^20^

#### General romantic relationship satisfaction

All participants completed the Relationship Assessment Scale (RAS), which measures satisfaction in intimate and romantic relationships. The RAS is a 7-item instrument quantified on a 5-point Likert scale ranging from 1 (strongly disagree) to 5 (strongly agree).^21^ It consists of questions such as “How well does your partner meet your needs” and “How many problems are there in your relationship?” (Appendix). We modified the questions to include the past tense for those reporting on previous relationships. The RAS generates a score between 7 and 35. A score between 7 and 17 indicates low satisfaction, 18–24 indicates moderate satisfaction, and 25–35 indicates high satisfaction.^21^ The RAS has a test–retest reliability of 0.85, as well as high concurrent validity with other common measures of relationship satisfaction; correlation coefficients range between 0.64 and 0.88 depending on the instrument used and the gender of the participants.^22^

**Table:** Relationship Assessment Scale Questions

	Strongly disagree				Strongly agree
How well does/did your partner meet your needs?	1	2	3	4	5
In general, how satisfied are/were you with your relationship?	1	2	3	4	5
How good is/was your relationship compared to most?	1	2	3	4	5
How often do/did you wish you hadn’t gotten into this relationship?	1	2	3	4	5
To what extent has/did your relationship met/meet your original expectations?	1	2	3	4	5
How much do/did you love your partner?	1	2	3	4	5
How many problems are/were there in your relationship?	1	2	3	4	5

#### Relational communication satisfaction

We developed the Relational Communication Satisfaction Scale (RCSS) as part of this project and used it only in conjunction with other measures. The RCSS differs from other relational satisfaction measures in that it requires each respondent to rate their partner’s communication and relational skills. This metric aids in understanding whether a partner’s neurotype can affect an autistic individual’s perception of their own social communication skills. The RCSS is modeled after the RAS and includes 10 items rated on the same 5-point Likert scale. It consists of statements such as “I feel/felt adequately supported by my partner” and “I am/was able to support my partner adequately” (Appendix). Five statements pertain to the respondent’s skills within the relationship, while the other five pertain to their partner’s skills.

**Table:** Relationship Communication Satisfaction Scale Statements

	Strongly disagree				Strongly agree
I feel that I am/was able to communicate my needs to my partner.	1	2	3	4	5
I feel that my partner is/was able to communicate their needs to me.	1	2	3	4	5
I am/was able to support my partner adequately.	1	2	3	4	5
I feel/felt adequately supported by my partner.	1	2	3	4	5
I am/was able to resolve conflicts with my partner effectively.	1	2	3	4	5
My partner is/was able to resolve conflict with me effectively.	1	2	3	4	5
I have had to adjust my communication style to fit my partner’s needs.	1	2	3	4	5
My partner has had to adjust their communication style to fit my needs.	1	2	3	4	5
My neurotype affects/affected my relationship with my partner.	1	2	3	4	5
My partner’s neurotype affects/affected our relationship.	1	2	3	4	5

#### Relationship-specific free-response questions

At the end of the survey, each participant answered three open-ended questions regarding the most positive and negative aspects of their current or past relationship and how they felt being autistic affected that relationship. The questions were as follows: (1) “Without using names or other identifying information, briefly describe the most positive aspects of your current/past relationship”; (2) “Without using names or other identifying information, briefly describe the most challenging/negative aspects of your current/past relationship”; (3) “Without using names or other identifying information, describe how you feel autism has affected your current/past relationship (in either positive or negative ways).”

### Data analysis

#### Quantitative analysis

To check for statistically significant differences between the SRS-2 scores of the professionally diagnosed and self-diagnosed individuals, we ran a pairwise *t-*test of equal variance using the sample mean scores from each group.^23^ This test used a significance level of *α* = 0.05 and 41 degrees of freedom. We also included effect sizes in our analysis, which can be interpreted as small (*d* < 0.2), medium (0.2 < *d* < 0.8), or large (*d* > 0.8).^24^

To analyze potential differences in relationship satisfaction, we conducted analysis of variance (ANOVA) tests to determine if there were statistically significant differences between cumulative RAS scores from A/A, A/NT, and A/ND dyads. If the ANOVAs resulted in statistical significance, we conducted post hoc pairwise *t*-tests to determine which dyad comparisons yielded statistically significant results. We repeated this process to analyze individual questions from the RAS and both the cumulative RCSS scores and the personal and partner skill ratings. Given the inclusion of participants who reported on past relationships (*n* = 17, 16.04%), we repeated these tests with only those who reported on current relationships and compared the results with the broader participant pool. In addition, because of the high number of ANOVAs run on the questions and statements from the RAS and RCSS, we used a Bonferroni correction to reduce the chances of a false positive. We did this by dividing the standard significance value (*α* = 0.05) by the number of ANOVAs run on the participant pool (12) resulting in a significance level of *α* = 0.004.^25^

#### Qualitative analysis

We analyzed the data using the six-phase approach to thematic analysis described by Willig and Rogers: (1) familiarization with the data, (2) creating the corresponding codes, (3) constructing themes based on the developed understanding and codes, (4) reviewing the generated themes, (5) defining the themes, and (6) producing the final report.^26^ We coded all data from A/A, A/NT, and A/ND dyads separately. We identified the most prominent and recurring themes from each group and compared and separated these into either positive or challenging factors for successful romantic relationships. To verify the generated themes, an additional coder reviewed 10% of the data, resulting in 83% agreement between the coder and the first author. We discussed and resolved the remaining discrepancies.

## Results

### Quantitative results

#### Social Responsiveness Scale, Second Edition

The SRS-2 data analysis revealed no statistically significant difference between the scores of self-diagnosed and professionally diagnosed participants (*α* = 0.05, *p =* 0.94). The similarity in mean scores indicated comparable self-reported levels of social differences. While there is a small risk that self-diagnosed individuals may fall outside the autism spectrum, answers from self-diagnosed individuals still contribute to understanding how relational satisfaction differs based on partners’ neurotypes. The inclusion of self-diagnosed participants allowed for a more comprehensive understanding of the autistic community, and the use of the SRS-2 limited the chance of incorrect self-identification.

#### Overall RAS scores

The results of the one-way ANOVA for the RAS scores indicated no significant difference between participant groups (*α* = 0.004, *p =* 0.11). The scores from each group indicated comparable levels of relationship satisfaction, irrespective of partner neurotype. While A/A (*x̄* = 29.13, *s* = 5.59) and A/ND (*x̄* = 28.52, *s* = 5.80) dyads demonstrated higher sample means when compared with A/NT (*x̄* = 25.89, *s* = 7.29) dyads, the differences were not statistically significant. The one-way ANOVA for the participants who reported on current relationships indicated similar results, with no significant difference between the three groups (*α* = 0.004, *p =* 0.108, *d* = 0.023).

#### Individual RAS response analysis

Out of the seven scaled statements, only one generated a statistically significant difference between two or more participant groups (*α* = 0.004, *p =* 0.0005, *d* = 0.145). The statement was “How much do/did you love your partner?” The pairwise *t-*tests determined that this question demonstrated statistically significant differences between A/A dyads and A/NT dyads and between A/ND and A/NT dyads, with participants in A/A and A/ND dyads reporting consistently higher scores when compared with those in A/NT dyads. When repeating these tests among only participants who reported on a current relationship, the results demonstrated no statistical difference across the three groups. In the case of the question, “How much do/did you love your partner?” the resulting *p*-value (*α* = 0.004, *p =* 0.012) did not meet the cutoff for the *α* postcorrection significance level.

#### RCSS scores

The results of the ANOVAs we ran on the combined RCSS scores indicated no statistically significant differences across the three participant pools (*α* = 0.004, *p =* 0.064). We found the same results when repeating this test only with participants who reported on current relationships (*α* = 0.004, *p =* 0.384). We also examined the five statements regarding personal skills and the five statements regarding partner skills and once again found there to be no significant differences across the three participant pools. We repeated this process for participants in current relationships, which again yielded statistically insignificant results.

The results of all ANOVAs are summarized in Table 2, and the results of the pairwise *t*-tests are summarized in Table 3. We adjusted both significance values to account for multiple statistical tests.^24^

**Table 2.** Analysis of Variance Results

	A/A		A/ND		A/NT		*p*-Value	α	Effect size
	Mean	SD	Mean	SD	Mean	SD			
Relationship Assessment Scale (RAS)									
Total RAS scores^a^	30.906	4.679	29.804	5.286	27.643	6.320	0.111	0.004	0.023
Question 1	3.969	1.204	3.848	1.010	3.500	1.139	0.241	0.004	0.011
Question 2	4.156	1.081	4.043	1.053	3.607	1.315	0.147	0.004	0.012
Question 3	4.406	0.946	4.130	1.087	3.857	1.079	0.132	0.004	0.005
Question 4	1.781	1.211	1.435	0.807	1.75	1.266	0.289	0.004	0.012
Question 5	3.938	1.105	3.609	1.273	3.571	1.425	0.442	0.004	0.005
Question 6	4.750	0.508	4.700	0.756	3.929	1.331	<0.001	0.004	0.145
Question 7	2.313	1.091	2.522	1.206	2.821	1.307	0.264	0.004	0.005
Relationship Communication Satisfaction Scale (RCSS)									
Total RCSS scores^b^	38.485	5.087	38.128	5.701	35.300	6.934	0.064	0.004	0.032
Self-rating statements^c^	19.515	2.884	19.638	2.937	17.767	4.125	0.038	0.004	0.041
Partner-rating statements^d^	18.970	2.531	18.489	3.161	17.533	3.329	0.167	0.004	0.015

a Cumulative RAS scores for each participant pool.

b Cumulative RCSS scores for each participant pool.

c Cumulative self-focused RCSS statements.

d Cumulative partner-focused RCSS statements.

SD, standard deviation.

**Table 3.** Pairwise *t-*Test Results: “How Much Do You Love Your Partner?”

Variable name	Group	Mean	Standard deviation	*p*-Value	Significance	Effect size
Comparison #1	A/A A/NT	4.750 3.929	0.508 1.301	0.004	0.017	0.908
Comparison #2	A/A A/ND	4.750 4.700	0.508 0.756	0.705	0.017	0.079
Comparison #3	A/ND A/NT	4.700 3.929	0.756 1.301	0.008	0.017	0.750

### Qualitative results

We identified a total of 15 themes from the three free-response questions (FRQs). There were seven themes relating to the most positive relational aspects, five themes relating to the most negative relational aspects, and three themes relating to how autism affected overall relationship satisfaction. In most cases, the themes were reported with similar frequency across the three participant pools, although there were some identifiable differences between the positive and negative themes, as well as in how participants reported the impact of autism on their relationships.

Given the intent toward understanding the impact of partner-neurotype on relationship satisfaction, we focus on the themes that demonstrated the most variance in terms of reporting based on each participant pool. All the identified themes with their associated subthemes are shown in Table 4.

**Table 4.** Free-Response Question Themes

Theme	Definition	Example(s)	Subthemes
Most positive aspects			
Acceptance and authenticity	*Acceptance and authenticity* is defined by a participant’s ability to be authentic in their relationship with no judgment of differences and no expectation for masking or hiding neurodivergent traits.	“I am able to fully un-mask around my husband without fear of judgment.” “Being able to be myself without being told it’s weird.”	No additional subthemes
Communication	*Communication* is defined by reported strengths in communication, conflict management, and discussing emotions/feelings.	“We can talk about anything, and I always feel comfortable bringing up problems even on sensitive subjects.”	Conflict resolution Honesty Good conversations Listening skills
Intimacy	*Intimacy* is defined by reports of both emotional and sexual and nonsexual physical intimacy as strengths within the relationships. These can include verbal and physical affection, as well as positive sexual experiences.	“I love cuddles, kisses and sex. I love waking up with my person next to me, and falling asleep next to them. I love loving, and feeling loved. I love having someone I can do nice things for. I love my partner’s voice, arms, laugh and smile. I love making them happy, and they make me happy.”	Emotional intimacy Physical intimacy Affection
General compatibility	*General Compatibility* is defined by an overall sense of compatibility. This encapsulates similarities in love language, humor, need for quality time, values, interests, and intellectual capabilities.	“He is incredibly sweet and considerate, very affectionate. We have a ton of fun together and have the same sense of humor. I love him deeply and we often discuss how compatible we are.”	Shared interests Shared values Shared humor and joy Quality time Intellectual similarities
Stability	*Stability* is defined by participants feeling secure and steady in their relationships. Reported relationships are built on foundations of trust, loyalty, lack of jealousy, and reliability.	“Our relationship is stable, loving, supportive, and overwhelmingly positive.” “How close we are and the love, trust, and respect we have for each other”	Relationship security Reliability Trust Loyalty Comfort
Support and accommodation	*Support and Accommodation* is defined by individuals feeling supported emotionally and in day-to-day life. Participants identified reciprocal support and the ability to accommodate differing needs within their relationships.	“She supports me and my autism very well, and I feel like I have the most fun with her than any other person. She is my best friend and makes me laugh like nobody else.”	No additional subthemes
Understanding	*Understanding* is defined by a deep and inherent sense of understanding between a participant and their partner. It focuses on a fundamental sense of understanding that does not require explanation. This is often attributed to shared neurodiversity within a relationship.	“We have a very good understanding of our unique ways of navigating relationships and don’t hold ourselves to societal standards of what we think a relationship should look like.”	No additional subthemes
Most challenging aspects			
Accommodating needs	*Accommodating Needs* is defined by challenges with supporting and/or meeting needs within the relationship (in a reciprocal sense). This can include meeting neurotype-specific needs, differences in support needs, accommodating differences in needs, and meeting emotional needs	“My partner has much higher support needs than I do which can be challenging at times when I can’t fulfill his needs, but we communicate about it regularly and try our best to make sure he gets the support he needs in other ways.”	Different support needs Sensory needs Emotional needs
Challenges attributed to neurotype	*Challenges Attributed to Neurotype* is defined by challenges that arise specifically due to the participants’ neurotypes, their partners’ neurotypes, or the interaction between differences in neurotypes within the relationships. Participants often reference traits commonly associated with autism, such as inflexibility, rigidity, sensory overwhelm, and meltdowns and shutdowns, as challenges within their relationships.	“We struggle to work around my inflexibility and rigidity due to my autism. Sometimes I don’t communicate effectively.” “Being in autistic burnout, I have difficulty completing daily tasks and this puts increased stress on my partner.”	Routines and rigidity Shutdowns and meltdowns Sensory overwhelm Autistic burnout Executive functioning Differing neurotypes
Communication challenges	*Communication Challenges* is defined by challenges primarily associated with general communication, the ability to communicate thoughts/feelings clearly, miscommunications, and conflict management/resolution.	“I wasn’t comfortable being in/resolving conflict and they had a hard time bringing up conflict in a way that I could engage with. I would try to respond to emotional problems with logic or support when that wasn’t what was being asked for, and more emotional support was being asked from me than I could give.”	Conflict resolution Communicating feelings and needs Misunderstandings (communication specific)
Emotional and mental health	*Emotional and Mental Health* is defined by challenges associated with emotional and mental health, such as anxiety, depression, self-esteem, fear of rejection, and other emotional challenges that may cause relational interference.	“My partner came in with a deeply anxious attachment, while I’m recovering from disorganized attachment. We’ve been able to make our way to a secure bond but it has been a ton of work! Autistic-allistic communication differences contributed some to that, but the biggest factor was past traumas playing into the present.”	Co-occurring mental health disorders: - Anxiety - Depression - Trauma/PTSD Rejection sensitivity Low self-esteem
Understanding and acceptance	*Understanding and Acceptance* is defined by challenges with understanding one another in terms of personal experiences, communication style (i.e., being “overly” emotional and/or logical), and needs. This category differs from *Communication Challenges* and *Accommodating Needs* because participants explicitly tied the source of their challenges back to an inherent lack of understanding between themselves and their partners.	“My husband tries for me to fit in, but my mental health is suffering from this (he wants me to mask/camouflage more than it is healthy for me to do. My mental health has taken a nosedive because of this. We are currently trying to find a balance between him being more accepting of outwardly autistic behavior vs. me masking to find balance that does not affect my mental health vs. him being embarrassed by me).”	No additional subthemes
Effect of autism			
Positive effect	Participants identify that autism positively affects the success and satisfaction of their relationships.	“Autism is one of the most important aspects of our relationship. I would not be able to have a relationship with someone who isn’t autistic, and it gives us an understanding of the other in a way that I would not be able to understand a neurotypical partner.”	No additional subthemes
Negative effect	Participants identify that autism negatively affects the success and satisfaction of their relationships.	“I can’t support her as well as I could because I shut down a lot and don’t like to talk about emotions. I also feel I struggle to communicate my feelings and thoughts, and I have had meltdowns where I have hit her before.”	No additional subthemes
Both positive and negative effects	Participants identify that autism both positively and negatively affects the success and satisfaction of their relationships.	“We are able to bond and express our special interests to each other where before our past relationships we didn’t have as much grace. We are able to understand each other’s sensory problems, and how severe and serious they can be. One of the more challenging things with autism is adulting in our relationships like marriage papers, taxes, mortgage/rent, etc etc. It’s hard for both of us to manage that.”	No additional subthemes

PTSD, post-traumatic stress disorder.

### Most positive aspects

#### Theme 1: Understanding

Understanding was a recurring theme primarily among A/A dyads. Across groups, participants often discussed the positive experience of being known by their partner, connecting through experiences, and not needing to justify or explain one’s thoughts or actions. Many participants, especially those with autistic partners, focused on how their shared neurodivergent identities fostered connection within their relationships.

Among those in A/A relationships, participants referred to an inherent understanding of their partners as one of the most important factors in the development and maintenance of the relationship:

“We really work together. We just click. We have a similar sense of humor and enjoy similar games and movies. But most importantly, we are on the same page about the value of our relationship and being teammates and partners” (A/A).

Some participants specifically discussed an appreciation for their shared neurotype in referring to how this allowed for a greater sense of mutual understanding: “I feel like we are able to understand how each other’s brain works and that helps a lot of issues, in the relationship and outside of the relationship” (A/A); “Our shared neurotype means that I can express myself in ways that are probably weirder than I would around most people. I can say things that don’t entirely make sense, but he understands” (A/A).

A/ND dyads shared perspectives with both respondents in A/A and A/NT dyads. Some participants reported that their partners understood aspects of them, such as communication style or need for personal time. One participant wrote: “For example, he does not ‘tone police’ me or demand that I state things in a certain way, he instead seeks to understand what I am trying to say” (A/ND). Others focused on a similar inherent sense of understanding as expressed by respondents in A/A dyads: “We ‘get’ each other better than others due to having similar ND traits (despite my partner being allistic)” (A/ND).

Members of A/NT dyads also referenced understanding as an important factor, but it was often characterized as trying to understand rather than a preexisting level of mutual understanding. One noted that their partner is “extremely supportive and tries to understand [their] experiences” (A/NT), which seems to differ from the inherent understanding described by many A/A dyads. Another wrote: “He also understands me better than most people and I think that’s because his brother is also autistic. He has tried to adapt his language to meet my needs” (A/NT). Several responses indicated that their partners needed to make a deliberate effort to understand their experiences.

#### Theme 2: Support and accommodation

Participants from all three groups reported that feeling supported by their partners contributed to the success of their relationships. Many also discussed the ability to provide support to their partners. Although members of A/A dyads mentioned support, most participants who discussed the roles of support in their relationships were in A/NT or A/ND dyads. Support as a positive aspect of the relationship was only mentioned on four occasions across A/A dyads.

One participant from an A/A dyad reported feeling supported during challenging moments: “My partner is very kind and gentle and patient, even when I am in a meltdown” (A/A). Another referenced this in relation to their shared autistic identity: “My partner made me feel seen, supported, and understood. I think they knew me and understood me remarkably well in our time together, probably because they were also autistic…” (A/A).

Respondents in A/ND dyads focused on the mutual ability to provide support: “I feel like we are a team, on the same side. We support each other with things that are hard for the other person” (A/ND); “We provide emotional and financial support to each other, especially when trying to achieve challenging long-term goals like career changes” (A/ND).

More than half of respondents in A/NT dyads mentioned support as an important positive quality of their relationships. One wrote: “He supports me at home by doing a majority of the chores that I struggle to do in a timely manner. He helps me keep a routine in regard to hygiene” (A/NT). Another participant noted that their partner provides “empathetic support when [they are] able to voice [their] needs” (A/NT).

#### Theme 3: Intimacy

Participants from all three dyads mentioned both physical (sexual and nonsexual) and emotional intimacy as primary strengths in their relationships. Interestingly, those with autistic partners reported intimacy far less often than those with non-autistic partners. Those with non-autistic partners generally put more emphasis on emotional intimacy. In particular, they cited feelings such as love, warmth, and closeness.

In the few cases where participants in A/A dyads discussed intimacy, they tended to focus on physical intimacy and affection as opposed to emotional intimacy. One wrote: “I could be physically close to my ex-partner very often as we both enjoyed being close to each other. We were both touch starved” (A/A). Others discussed “physical attraction” and “physical affection” as positive aspects of their relationships among other things. Only one participant cited emotional intimacy as a primary positive factor.

Respondents in A/ND dyads tended to focus more on emotional intimacy as a strength in their relationships. One respondent wrote: “Our relationship is stable, loving, supportive, and overwhelmingly positive” (A/ND). Another reported “love and kindness, living together peacefully, getting along on a day to day basis, [and] commitment/length of relationship” (A/ND) as the most important positive factors.

One participant discussed both emotional and physical intimacy, writing “he is incredibly sweet and considerate, very affectionate. We have a ton of fun together and have the same sense of humor. I love him deeply and we often discuss how compatible we are” (A/ND).

Participants in A/NT dyads also discussed emotional intimacy, with multiple individuals reporting the love within their relationships as cornerstones of their relationships. One participant wrote: “We enjoy our time together, we are emotionally intimate, I feel safe and can be vulnerable with him” (A/NT). Another reported on both physical and emotional intimacy:

I love cuddles, kisses and sex. I love waking up with my person next to me, and falling asleep next to them. I love loving, and feeling loved. I love having someone I can do nice things for. I love my partner’s voice, arms, laugh and smile. I love making them happy, and they make me happy. (A/NT)

#### Theme 4: Authenticity/acceptance

When discussing the most positive aspects of their relationships, participants referenced the ability to be authentic without feeling judged by their partners. Feelings of acceptance were discussed in comparable amounts across all groups, with slightly more occurrences among A/A dyads.

Respondents in A/A dyads expressed their appreciation for mutually understood authenticity: “We can sit in the same room each doing our own thing and not [make] eye contact and still feel close. We understand and celebrate each other’s special interests” (A/A). One participant wrote that the most positive aspect of their relationship was “being able to have big feelings and not be judged, truly being able to be myself with another person” (A/A).

Participants in A/ND dyads discussed themes akin to those in A/A dyads: “We’re very open to stimming as needed…” (A/ND), referring to the shared ability to engage in self-stimulatory behavior. Another discussed reciprocal acceptance in their relationship, noting each partner’s strengths and weaknesses:

I think we have created a space where the other feels accepted for who they are, even if this means our fights look different or our communication needs are different. We also don’t shame each other for not doing things the way others expect us to, and instead try to support each other the best we can. (A/ND)

Some respondents in A/NT dyads reported similar anecdotes with one stating: “I am able to fully un-mask around my husband without fear of judgment” (A/NT). Another reported feeling valued for their differences: “I felt accepted for who I was and my quirks were appreciated” (A/NT).

### Most negative aspects

#### Theme 1: Communication

While some reported communication to be a positive characteristic, respondents generally expressed feeling challenged by how communication affected the quality of their relationships.

A/A dyads reported the fewest number of communication issues, but nearly half of those respondents still listed communication—general challenges, conflict issues, or miscommunications—as one of the most difficult relational aspects. One individual wrote that “communication issues, shutting down, and holding things in until we blew up” (A/A) were the most negative parts of their relationship. Others specifically referenced challenges with conflict avoidance and resolution:

Sometimes it’s hard to figure out how to talk about difficult subjects. Sometimes my partner shuts down or avoids confronting things that are bothering him rather than telling me so we can solve the problem and then the problem gets bigger. (A/A)

Similar to A/A dyads, members of A/ND dyads focused on how their respective neurodiversities contributed to creating challenges with miscommunication:

Sometimes I over-assume she is being completely literal/honest/forthcoming when she’s not and sometimes she assumes I’m being nonliteral/subtle when I’m not. We consistently have to try to remember that the other person communicates in a different way. But I think we are doing a really good job of it. (A/ND)

Participants in A/NT dyads frequently reported difficulty communicating with their partners in general; one person attributed these challenges to the inherent differences between themself and their partner: “…we simply think about things so incredibly differently that it makes it hard to communicate our thoughts and this leads to tension” (A/NT). Some respondents focused on difficulties with communicating feelings: “I am unable to communicate my emotional needs to my husband” (A/NT). Participants in this group also mentioned times when they felt their communication was inadequate: “Communicating when I’m mute, my sudden anger when in environments I can’t regulate or control (grocery stores, long trips, etc.)” (A/NT).

#### Theme 2: Challenges attributed to differences in neurotype

When reporting the most challenging aspects of their relationships, many participants noted struggling to navigate inherent differences between themselves and their partners. Respondents referenced personal characteristics, such as rigidity and inflexibility, as well as experiences with shutdowns and sensory overload as factors that created obstacles in their relationships.

Participants from A/A dyads focused on how experiencing shutdowns and meltdowns posed challenges in their relationships. One wrote: “Sometimes my partner shuts down or avoids confronting things that are bothering him rather than telling me so we can solve the problem and then the problem gets bigger” (A/A). Another focused on how contrasting characteristics within their relationships created obstacles: “I have a tendency to get angry when my routines are disrupted or when things aren’t being done to my satisfaction. I tend to get upset when he doesn’t follow my direction, and he shuts down…” (A/A).

Many participants in A/ND dyads wrote about obstacles stemming from the interactions between autism and other forms of neurodiversity. One participant focused on how their partner’s ADHD contradicted their need for routine: “Time management is by far the hardest aspect. I am a very punctual person and get very frustrated when my schedule changes and my partner’s ADHD makes it super hard for them to be on time. It’s difficult to navigate” (A/ND). One participant shared discomfort with their partner’s challenges with maintaining clean spaces:

My spouse has ADHD so she is chaotic and I need lots of order. This affects many aspects of our relationship. I need cleanliness and order while my spouse is very messy. I can’t think clearly around clutter and messiness and it makes me nervous. (A/ND)

Individuals in A/NT dyads reported feeling challenged by the differences between themselves and their partners, with some attributing this directly to being autistic: “We struggle to work around my inflexibility and rigidity due to my autism” (A/NT). One participant discussed feeling inadequate due to not being neurotypical: “I feel as though I cannot do many things that a neurotypical person would be able to do to support my husband” (A/NT).

### Perceived effects of autism on the relationship

When respondents were asked to comment on how their autistic traits played a role in their relationships, more than half of the individuals in A/A dyads reported that being autistic benefited their relationships. One participant described how autism made them feel more connected with their partners:

Autism is one of the most important aspects of our relationship. I would not be able to have a relationship with someone who isn’t autistic, and it gives us an understanding of the other in a way that I would not be able to understand a neurotypical partner. (A/A)

Another expressed a similar sentiment by focusing on their shared interests and ability to accommodate one another:

We are able to bond and express our special interests to each other where before our past relationships we didn’t have as much grace. We are able to understand each other’s sensory problems, and how severe and serious they can be. (A/A)

Members of A/A dyads were the most likely to share positive anecdotes about autism in the context of their relationships out of the three participant pools, although some still reported that autism primarily created obstacles: “We both are two different ends of autism (myself logical and them emotional) and it means we sometimes struggle when having a meltdown to comfort the other” (A/A).

A/ND dyads frequently reported that being autistic posed more challenges than benefits in their relationships: “I feel like I’m not always able to contribute equally, which makes me feel guilty, then I feel insecure about my position in the relationship” (A/ND). Multiple participants described how they felt autism had negatively affected their communication skills, with one noting that being autistic posed the most obstacles when navigating conflict: “It has mostly affected our disagreements. It also causes problems when someone wants to make spontaneous plans or when someone we are meeting up with is late” (A/ND). Another discussed their broader relationship experiences: “I am not good at social cues and picking up hints, so I often hurt people’s feelings when I am trying to help” (A/ND); “I don’t pick up on or enjoy discussing/expressing emotions as much as neurotypicals, sensory overwhelm, frustration on inconsistencies, burnout from social demands” (A/ND).

Despite the number of individuals who viewed autism as an obstacle in their relationships, one focused on how they felt neurodiversity brought them closer to their partner: “Us both being neuroatypical has been a positive, we see the world in a similar way, are comfortable in ourselves, and bond over our similarities” (A/ND). Another wrote about how being autistic improved their communication and commitment: “This is just me, but I feel like it makes me a more committed partner because I am always consciously thinking about how to overcome communication challenges and therefore put more effort into this than the average person” (A/ND).

A majority of participants in A/NT dyads reported that they felt being autistic had created challenges in their relationships. Many members of A/NT dyads spoke about how personal struggles negatively affected their relationships: “My autism causes extra stress for my partner as he has to pick up the slack in terms of chores” (A/NT); “My husband has to put up with meltdowns, irritability, [and] my need for control over situations” (A/NT). Over half of the participants from the A/NT dyads reported strictly negative experiences related to how autism affected their relationships. In contrast, only two individuals from the A/NT dyads wrote that autism created a strictly positive impact:

I’m very organized and run a three child household well. As we have an autistic child I am a good resource for speaking up for him and navigating his schooling needs. My partner is an introvert although neurotypical so we both choose home life over socializing but he is still more outgoing than I am. (A/NT)

Some shared both positive and negative relationship experiences related to being autistic:

I think my autism helped me get interested in some of the interests of my partner by turning them into special interests once I had been introduced to them. But also my autism can cause a lot of misunderstandings because I do not always understand what my partner is trying to communicate to me, since they do not automatically communicate in a direct manner. (A/NT)

## Discussion

We intended to expand upon existing literature regarding romantic relationship satisfaction for autistic adults by exploring the potential impact of partner neurotype. The results of the RAS indicated that respondents found comparable levels of romantic satisfaction, irrespective of partner neurotype. Despite relative consistency in overall satisfaction, members of A/A and A/ND dyads endorsed that they love their partners more, which may be a factor in the trend of slightly higher sample mean satisfaction reported by respondents in A/A and A/ND dyads. However, the sample means from each group fell within the range indicative of high levels of romantic relationship satisfaction (=25–35), affirming that autistic individuals are able to establish and thrive in successful romantic partnerships across a broad range of neurotypes. These findings contradict previous conclusions stating that autistic individuals are most likely to find satisfaction with autistic partners,^2^ as well as the implication that they are less capable of maintaining satisfying romantic relationships with other neurotypes. The resulting RCSS scores further verified these findings, with individuals across all three participant pools indicating comparably high levels of satisfaction with reciprocal communication within their relationships. Regardless of partner neurotype, many autistic individuals appear able to navigate relationship challenges and find fulfillment with autistic and non-autistic partners alike.

*Understanding* was a key theme underpinning a lot of A/A relationships. This makes sense given the strong likelihood of preexisting, mutually shared values and experiences inherent when both partners possess an autistic identity. *Understanding* takes on a different meaning within a relationship of differing neurotypes, as each partner may not have an inherent sense of the other person’s lived experience, opinions, or preferences. It is an active process of discovery requiring a desire to recognize and appreciate different approaches to communicating and dealing with life’s challenges. It is important to note that autistic individuals are not unique in wanting to feel understood by their partners. Regardless of diagnostic status, preexisting research demonstrates that a reciprocal sense of understanding in a relationship predicts high levels of trust, intimacy, and adjustment skills.^27,28^

The results of the FRQs highlighted that feeling understood by a partner was one of the most endorsed positive factors for A/A dyads. Respondents emphasized the importance of a deep and inherent sense of understanding from their partners, which was often attributed to their shared autistic identities. This theme was not as commonly reported by those in A/NT or A/ND dyads. Instead, it was functionally replaced by the positive impact of feeling supported and accommodated by a partner. Participants in both A/NT and A/ND dyads discussed how day-to-day support from their partners helped contribute to strong and fulfilling relationships. Although *support* was not frequently mentioned by participants in A/A dyads, the results of the RCSS indicated that respondents from all groups found that their partners were comparably capable of providing adequate support. This implies that *understanding* itself may function as a form of *support*, making it an important factor to consider in the context of romantic relationship success for autistic populations.

Both physical intimacy and emotional intimacy were reported as strengths within neurodiverse relationships. There were differences in the volume of reporting based on partner neurotype, with those in A/A dyads focusing less on emotional intimacy. To draw conclusions from this finding, we would need to do further research detailing the role of intimacy in A/A relationships.

Participants commonly discussed feelings of *acceptance* and *authenticity* as positive contributors to relationship satisfaction. Unlike those in A/A and A/ND dyads, participants in A/NT dyads expressed appreciation for how their partners created a safe space for authenticity. Those in A/A dyads were more likely to discuss the shared space for mutual acceptance. While this is a small difference in expression, it conveys a unique quality of neurodiverse relationships that may present differently in A/NT dyads. The appreciation for acceptance also demonstrates a potential barrier for autistic individuals when pursuing romantic relationships, as this quality may be more difficult to find in neurotypical partners.

*Communication* was frequently referenced when discussing the most challenging relational aspects. This was unsurprising, as social-communication differences are a key trait of an autism diagnosis.^29^ With that said, autistic individuals are not the only ones to report communication as a challenge in their relationships. Communication difficulties are one of the most common predictors of relationship dissatisfaction and termination.^30^ The high frequency of communicative challenges specifically among A/NT and A/ND relationships is aligned with the double-empathy problem, which indicates increased levels of connectedness between autistic individuals.^31^ Despite this, communication still proved to be a significant challenge for A/A dyads, despite their shared neurotype. Given the positive relationship between communicative skills and relationship satisfaction,^15,32^ it is important to further develop current support practices to include the needs of autistic relationships.

All three groups expressed that communication felt like an obstacle, but members from each dyad had different interpretations as to why. Individuals in A/A and A/ND dyads tended to reference communicative issues in the relationship, while those in A/NT focused more on how their personal struggles with communication created challenges for their partners. This difference in perspective suggests that social communication differences commonly attributed to an autism diagnosis may be less accepted by neurotypical partners, which reveals a potential area of vulnerability within A/NT relationships. Although A/ND dyads may not have demonstrated the same levels of inherent understanding as A/A dyads, it is possible that the shared experience of neurodiversity (and miscommunication) led to higher levels of empathy when encountering relationship-specific communication differences.

In addition to communication challenges, some participants specifically expressed that behavioral and cognitive characteristics associated with an autism diagnosis, such as meltdowns, sensory overload, and rigidity, were the primary source of challenges in their relationships. It was common for individuals in A/NT dyads to describe their autism as a personal shortcoming, while those in A/A dyads tended to focus on their shared autistic traits as obstacles that both partners faced together. Participants in A/ND dyads shared similar sentiments but focused more on neurodiversity as a whole. Individuals in A/A dyads, despite feeling challenged at times by the shared autism diagnosis, generally had very positive feelings about how autism affected their relationships. Many referred to the sense of inherent understanding they shared with their partners and discussed the ways in which they connected due to mutual experiences. Both findings suggest that the neurotype of an autistic individual’s partner may directly influence their perception and opinion of autism, which could contribute to previously reported relationship satisfaction discrepancies when comparing autistic and non-autistic partners.^2–4^

### Limitations and future directions

While this investigation has yielded important insights, there are limitations that should be addressed in future research. There were a low proportion of participants identifying as male, which means that these findings may be more applicable to women and nonbinary individuals. Also, we did not collect information on potential co-occurring diagnoses from participants. It is uncertain if additional secondary diagnostic conditions were present that could potentially influence the resulting themes.

Another limitation is the potential inaccuracy of reported partner neurotypes, as we did not verify them across all participants. In addition, we did not obtain satisfaction ratings and question responses from participant partners, which would enable us to examine if reported relationship satisfaction and endorsements were comparable between relationship partners. Finally, because of the elevated levels of relationship satisfaction endorsed across participant groups, it is possible that motivation to participate in this study stemmed from unusually positive relationship experiences and perspectives. It is possible that autistic individuals with relatively low levels of relationship satisfaction or multiple failed relationships were less inclined to participate. If true, this could make the results less representative of the broader autistic population.

It would be interesting (although more challenging for recruitment) for future studies to include both members of each dyad to be able to explore potential converging and diverging themes and levels of relationship satisfaction. There is also a specific need to explore how communication needs differ among autistic individuals in romantic relationships. This follow-up investigation could be done through a series of interviews involving autistic participants and their partners, with the questions aimed at examining specific instances of communication-related success and failure. The goal of such a study would be to understand how to best support autistic individuals in a way that is empowering and leverages their own strengths and communication style instead of attempting to teach adherence to non-autistic standards. By identifying where differences in communication (and underlying experience) arise and seeing how these contribute to healthy and fulfilling relationships, current clinical practices can be improved to support people based on the described characteristics that lead to success. Such work could also aid in developing resources for autistic individuals who may lack confidence in pursuing relationships by focusing on identifying their strengths and leveraging their unique understanding of the world. The overarching intent is to adjust the current definition and perception of a successful relationship to include the lived experience of autistic individuals.

## Conclusion

Our study indicates that the endorsed positive and challenging factors within a successful relationship appear to differ based on partner neurotype, but overall relationship satisfaction remains high across all dyad combinations. Furthermore, the neurotype of an autistic individual’s partner directly influences their perception of autism’s impact, as participants in A/A dyads tended to feel more positively about how being autistic affected their relationships, while respondents from A/NT and A/ND tended to feel more negatively. Given the link between high levels of self-esteem and increased relationship fulfillment,^33^ this may explain the overall satisfaction discrepancies observed when comparing autistic and non-autistic individuals as seen in previous studies.^2^ It is likely that the factors contributing to romantic relational satisfaction are influenced by the presence, or absence, of shared experiences related to being autistic.

It is important to note that while the enabling and barring relationship factors differed based on partner neurotype, the purpose of this project was not to identify a specific neurotype combination associated with the highest overall relationship satisfaction, nor was the intention to rank the satisfaction level of different neurotype dyads. Instead, the intention is to better understand how autistic individuals can thrive and best be supported when navigating romantic partnerships by identifying the unique factors identified as key contributors to strong, healthy relationships with diverse partner neurotypes.

This study also serves as a reminder that autistic individuals are not a homogenous community. Despite their shared neurotype, the experiences of autistic individuals in romantic relationships are not shared. Having an autistic identity does not predetermine one’s ability or desire to pursue relationships with others. As with all neurotypes, the success or failure of a romantic relationship is due to myriad factors and cannot be solely attributed to or predicted by an individual’s diagnostic status. Autism can potentially affect romantic relationship duration and satisfaction,^2–4^ but it is one of many factors to consider when learning how to support individuals in pursuing and maintaining successful relationships. Instead, the whole relationship needs to be explored, just as is done when finding the strengths and weaknesses of non-autistic relationships. Although specific relationship needs may differ based on partner neurotype, it is clear that autistic individuals can find fulfillment with autistic, neurodivergent, *and* neurotypical partners.

## References

[1] Yew RY, Samuel P, Hooley M, Mesibov GB, Stokes MA. A systematic review of romantic relationship initiation and maintenance factors in autism. Personal Relationships. 2021; 28(4):777–802; doi: 10.1111/pere.12397

[2] Strunz S, Schermuck C, Ballerstein S, Ahlers CJ, Dziobek I, Roepke S. Romantic relationships and relationship satisfaction among adults with Asperger syndrome and high-functioning autism. J Clin Psychol. 2017; 73(1):113–125; doi: 10.1002/jclp.22319 27196958

[3] Hancock G, Stokes MA, Mesibov G. Differences in romantic relationship experiences for individuals with an autism spectrum disorder. Sex Disabil. 2020; 38(2):231–245; doi: 10.1007/s11195-019-09573-8

[4] Barneveld PS, Swaab H, Fagel S, van Engeland H, de Sonneville LMJ. Quality of life: A case-controlled long-term follow-up study, comparing young high-functioning adults with autism spectrum disorders with adults with other psychiatric disorders diagnosed in childhood. Compr Psychiatry. 2014; 55(2):302–310; doi: 10.1016/j.comppsych.2013.08.001 24290884

[5] Billstedt E, Gillberg IC, Gillberg C. Autism in adults: Symptom patterns and early childhood predictors. Use of the DISCO in a community sample followed from childhood. J Child Psychol Psychiatry. 2007; 48(11):1102–1110; doi: 10.1111/j.1469-7610.2007.01774.x 17995486

[6] Eaves LC, Ho HH, Eaves DM. Subtypes of autism by cluster analysis. J Autism Dev Disord. 1994; 24(1):3–22; doi: 10.1007/bf02172209 8188572

[7] Orsmond GI, Krauss MW, Seltzer MM. Peer relationships and social and recreational activities among adolescents and adults with autism. J Autism Dev Disord. 2004; 34(3):245–256; doi: 10.1023/b:jadd.0000029547.96610.df 15264493

[8] Orsmond GI, Shattuck PT, Cooper BP, Sterzing PR, Anderson KA. Social participation among young adults with an autism spectrum disorder. J Autism Dev Disord. 2013; 43(11):2710–2719; doi: 10.1007/s10803-013-1833-8 23615687 PMC3795788

[9] Stokes M, Newton N, Kaur A. Stalking, and social and romantic functioning among adolescents and adults with autism spectrum disorder. J Autism Dev Disord. 2007; 37(10):1969–1986; doi: 10.1007/s10803-006-0344-2 17273936

[10] Mintah K, Parlow SE. Are you flirting with me? Autistic traits, theory of mind, and inappropriate courtship. Personality and Individual Differences. 2018; 128:100–106; doi: 10.1016/j.paid.2018.02.028

[11] Kohn BH, Vidal P, Chiao R, Pantalone DW, Faja S. Sexual knowledge, experiences, and pragmatic language in adults with and without autism: Implications for sex education. J Autism Dev Disord. 2023; 53(10):3770–3786; doi: 10.1007/s10803-022-05659-z 35922688 PMC9362056

[12] Braithwaite S, Holt-Lunstad J. Romantic relationships and mental health. Curr Opin Psychol. 2017; 13(13):120–125; doi: 10.1016/j.copsyc.2016.04.001 28813281

[13] Hudson CC, Hall L, Harkness KL. Prevalence of depressive disorders in individuals with autism spectrum disorder: A meta-analysis. J Abnorm Child Psychol. 2019; 47(1):165–175; doi: 10.1007/s10802-018-0402-1 29497980

[14] Hollocks MJ, Lerh JW, Magiati I, Meiser-Stedman R, Brugha TS. Anxiety and depression in adults with autism spectrum disorder: A systematic review and meta-analysis. Psychol Med. 2019; 49(4):559–572; doi: 10.1017/s0033291718002283 30178724

[15] Du Plooy K, de Beer R. Effective interactions: Communication and high levels of marital satisfaction. Journal of Psychology in Africa. 2018; 28(2):161–167; doi: 10.1080/14330237.2018.1435041

[16] Crossley A, Langdridge D. Perceived sources of happiness: A network analysis. J Happiness Stud. 2005; 6(2):107–135; doi: 10.1007/s10902-005-1755-z

[17] Dush CMK, Amato PR. Consequences of relationship status and quality for subjective well-being. Journal of Social and Personal Relationships. 2005; 22(5):607–627; doi: 10.1177/0265407505056438

[18] Taboas A, Doepke K, Zimmerman C. Preferences for identity-first versus person-first language in a US sample of autism stakeholders. Autism. 2023; 27(2):565–570; doi: 10.1177/13623613221130845 36237135

[19] Buijsman R, Begeer S, Scheeren AM. “Autistic person” or “person with autism”? Person-first language preference in Dutch adults with autism and parents. Autism. 2022; 27(3):136236132211179; doi: 10.1177/13623613221117914 PMC10074744 35957517

[20] Bruni TP . Test Review: Social Responsiveness Scale–Second Edition (SRS-2). Journal of Psychoeducational Assessment. 2014; 32(4):365–369; doi: 10.1177/0734282913517525

[21] Hendrick SS, Hendrick C, Adler NL. Romantic relationships: Love, satisfaction, and staying together. Journal of Personality and Social Psychology. 1988; 54(6):980–988; doi: 10.1037/0022-3514.54.6.980

[22] Hendrick SS, Dicke A, Hendrick C. The Relationship Assessment Scale. Journal of Social and Personal Relationships. 1998; 15(1):137–142; doi: 10.1177/0265407598151009

[23] Xu M, Fralick D, Zheng JZ, Wang B, Tu XM, Feng C. The differences and similarities between two-sample t-test and paired t-test. Shanghai Arch Psychiatry. 2017; 29(3):184–188; doi: 10.11919/j.issn.1002-0829.217070 28904516 10.11919/j.issn.1002-0829.217070 PMC5579465

[24] Cohen J . *Statistical Power Analysis for the Behavioral Sciences* . 2nd ed. Routledge; 1988; doi: 10.4324/9780203771587

[25] Haynes W . Bonferroni Correction. Encyclopedia of Systems Biology. 2013; 154–154; doi: 10.1007/978-1-4419-9863-7_1213

[26] Willig C, Rogers WS. *The SAGE Handbook of Qualitative Research in Psychology* . 2nd ed. Sage Publications; 2017.

[27] Pollmann MMH, Finkenauer C. Investigating the role of two types of understanding in relationship well-being: Understanding is more important than knowledge. Pers Soc Psychol Bull. 2009; 35(11):1512–1527; doi: 10.1177/0146167209342754 19638635

[28] Reis HT, Lemay EP, Finkenauer C. Toward understanding understanding: The importance of feeling understood in relationships. Social & Personality Psych. 2017; 11(3):e12308; doi: 10.1111/spc3.12308

[29] American Psychiatric Association. *Diagnostic and Statistical Manual of Mental Disorders* . 5th ed. American Psychiatric Publishing; 2013; doi: 10.1176/appi.books.9780890425596

[30] Markman HJ, Rhoades GK, Stanley SM, Ragan EP, Whitton SW. The premarital communication roots of marital distress and divorce: The first five years of marriage. J Fam Psychol. 2010; 24(3):289–298; doi: 10.1037/a0019481 20545402 PMC4298140

[31] Botha M, Dibb B, Frost DM. “Autism is me”: An investigation of how autistic individuals make sense of autism and stigma. Disability & Society. 2022; 37(3):427–453; doi: 10.1080/09687599.2020.1822782

[32] Eğeci İS, Gençöz T. Factors associated with relationship satisfaction: Importance of communication skills. Contemp Fam Ther. 2006; 28(3):383–391; doi: 10.1007/s10591-006-9010-2

[33] Harris MA, Orth U. The link between self-esteem and social relationships: A meta-analysis of longitudinal studies. J Pers Soc Psychol. 2020; 119(6):1459–1477; doi: 10.1037/pspp0000265 31556680

